# Evaluating methods for measuring background connectivity in slow event‐related functional magnetic resonance imaging designs

**DOI:** 10.1002/brb3.3015

**Published:** 2023-04-16

**Authors:** Lea E. Frank, Dagmar Zeithamova

**Affiliations:** ^1^ Department of Psychology University of Oregon Eugene Oregon USA

**Keywords:** background connectivity, connectivity fingerprint, individual differences, resting‐state functional connectivity, task‐based functional connectivity

## Abstract

**Introduction:**

Resting‐state functional magnetic resonance imaging (fMRI) is widely used for measuring functional interactions between brain regions, significantly contributing to our understanding of large‐scale brain networks and brain–behavior relationships. Furthermore, idiosyncratic patterns of resting‐state connections can be leveraged to identify individuals and predict individual differences in clinical symptoms, cognitive abilities, and other individual factors. Idiosyncratic connectivity patterns are thought to persist across task states, suggesting task‐based fMRI can be similarly leveraged for individual differences analyses.

**Method:**

Here, we tested the degree to which functional interactions occurring in the background of a task during slow event‐related fMRI parallel or differ from those captured during resting‐state fMRI. We compared two approaches for removing task‐evoked activity from task‐based fMRI: (1) applying a low‐pass filter to remove task‐related frequencies in the signal, or (2) extracting residuals from a general linear model (GLM) that accounts for task‐evoked responses.

**Result:**

We found that the organization of large‐scale cortical networks and individual's idiosyncratic connectivity patterns are preserved during task‐based fMRI. In contrast, individual differences in connection strength can vary more substantially between rest and task. Compared to low‐pass filtering, background connectivity obtained from GLM residuals produced idiosyncratic connectivity patterns and individual differences in connection strength that more resembled rest. However, all background connectivity measures were highly similar when derived from the low‐pass‐filtered signal or GLM residuals, indicating that both methods are suitable for measuring background connectivity.

**Conclusion:**

Together, our results highlight new avenues for the analysis of task‐based fMRI datasets and the utility of each background connectivity method.

## Graphical abstract

In this manuscript, we measured the similarity of resting‐state connectivity profiles to those obtained during slow event‐related functional magnetic resonance imaging (fMRI). Importantly, background connectivity relies on the removal of task‐evoked signals to isolate intrinsic functional interactions. Here, we compared two common methods for removing task‐evoked activity: (1) applying a low‐pass filter to the fMRI time‐series signal, or (2) extracting the residuals from a general linear model of task‐evoked responses.

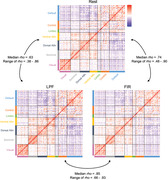

## 1 INTRODUCTION

Neuroimaging studies have revealed that distant brain regions can exhibit correlated neural activity, or “functional connectivity,” even in the absence of external stimulation (Clark et al., 1984; Friston, 1994, 1993; Shaw, 1981; Tucker et al., 1986). The most common approach to measuring functional connectivity is with resting‐state functional magnetic resonance imaging (fMRI), during which spontaneous low‐frequency fluctuations in brain activity are recorded in the absence of an explicit task. Resting‐state functional connectivity studies have substantially contributed to our understanding of brain organization and brain–behavior relationships. A key contribution of resting‐state connectivity studies has been the identification of large‐scale brain networks (Power et al., 2012; Wang et al., 2015; Yeo et al., 2011). These functional networks align with what we know from task‐based fMRI (Beckmann et al., 2005; Dosenbach et al., 2007; Fox, Corbetta, et al., 2006; Greicius et al., 2003) and are relatively stable across time and individuals (Gratton et al., 2018; Guo et al., 2012; Horien et al., 2019; Zuo et al., 2010). Although functional connectivity does not imply existence of underlying structural connections, regions that are structurally connected tend to exhibit high functional connectivity (Greicius et al., 2009; Honey et al., 2007; Passingham et al., 2002; Rykhlevskaia et al., 2008; Van Den Heuvel et al., 2009).

While network structure is relatively consistent across individuals, functional connectivity measures also contain important information about individual differences. For example, stable idiosyncratic differences exist among individuals such that a pattern of an individual's connections may serve as their “connectivity fingerprint” (Finn et al., 2015). Furthermore, a range of studies have demonstrated the functional relevance of individual differences in connectivity, such that the strength of a specific connection or a network metric can predict individual differences in clinical (Reinen et al., 2018; Takamura & Hanakawa, 2017; Tracy & Doucet, 2015), cognitive (Finn et al., 2015; Fong et al., 2019; Rosenberg et al., 2015), or age variables (Dosenbach et al., 2010; Ferreira & Busatto, 2013; Geerligs et al., 2015; Wang et al., 2012).

While resting‐state connectivity measures remain the gold standard, follow‐up work has also explored the utility of connectivity measures obtained while a person performs a task. A frequently utilized functional connectivity measure in task‐based designs is *background connectivity*, or coupling between regions that is observed after task‐evoked activity has been controlled for (Al‐Aidroos et al., 2012; Fair et al., 2007; Frank, Bowman, et al., 2019; Frank, Preston, et al., 2019; Norman‐Haignere et al., 2012). Such measures can provide information about how interactions between regions are altered by different cognitive demands, for example, comparing background connectivity under different task conditions (Al‐Aidroos et al., 2012; Cooper & Ritchey, 2019; Norman‐Haignere et al., 2012; Tambini et al., 2017). Notably, some argue that connectivity patterns found during rest persist during different task states, with task‐evoked activity comprising a modest proportion of overall connectivity structure (Cole et al., 2014; Gratton et al., 2018; Kraus et al., 2021). As Fair et al. (2007) suggested, one may not need to collect a dedicated rest scan; it may be possible to extract resting‐state‐like connectivity profiles from task‐based fMRI, to identify network structure and measure individual differences in connectivity. With the growth of publicly available magnetic resonance imaging (MRI) data, this means researchers can get further use out of task‐based fMRI datasets.

A key challenge with measuring functional connectivity in task‐based designs is the potential confounding role of task‐related activity. For example, two regions that are otherwise minimally functionally connected may both show task‐evoked activity, such as both increasing activation in response to stimulus onset. Correlating raw activation time courses would generate conflated connectivity estimates that do not reflect the true nature of their functional relatedness. Yet, if brain activity during a task is a roughly linear combination of spontaneous and task‐evoked activations—as has been suggested (Fox & Raichle, 2007; Fox, Snyder, et al., 2006)—it may be possible to isolate resting‐state‐like connectivity profiles after statistically removing task‐evoked activity from the observed signal (Cole et al., 2014; Fair et al., 2007; Gratton et al., 2018).

The degree to which background connectivity may approximate resting‐state connectivity is, however, not yet clear. For example, Fair and colleagues (2007) showed that background connectivity estimates obtained from block‐design task‐based fMRI produced average connection patterns similar to those found at rest. In contrast, larger differences compared to rest were found when extracting background connectivity from a jittered event‐related fMRI design. Furthermore, how “connectivity fingerprints” or individual differences in background connectivity relate to those from resting‐state could not be evaluated, as the study by Fair and colleagues measured background connectivity and resting‐state connectivity in different subjects.

Another question is how the correspondence between rest and background connectivity estimates may be affected by a specific method of removing task‐evoked responses. For example, Fair and colleagues (2007) modeled task‐evoked responses with a general linear model (GLM), using a finite impulse response (FIR) function to maximize the fit between the blood‐oxygen‐level‐dependent(BOLD) signal and the model (Al‐Aidroos et al., 2012; Cooper & Ritchey, 2019; Duncan et al., 2014; Norman‐Haignere et al., 2012). After the task‐evoked signal was modeled out, background connectivity was then computed using the residual time series. Another approach, suited for slow event‐related designs with regularly spaced trials, is applying a low‐pass filter to remove activity fluctuations at the task frequency and leaving only lower frequency fluctuations reflecting background activity (Frank, Bowman, et al., 2019; Frank, Preston, et al., 2019; Tambini et al., 2017). While applying a low‐pass filter can be a computationally faster alternative to FIR modeling, it is unclear whether the two methods produce similar connectivity profiles and how they each compare to rest.

Here, we tested the idea that resting‐state‐like connectivity may be obtained from task‐based fMRI after the removal of task‐evoked signals, by measuring the congruency between background connectivity estimates and resting state‐connectivity in a slow event‐related fMRI design. We focused on slow event‐related fMRI as it has not been formally compared to rest, and its use may be increasing due to its benefits for trial‐by‐trial multivariate pattern analyses (Zeithamova et al., 2017). Moreover, a slow event‐related design allows for both methods of removing task‐evoked activation: FIR modeling (Al‐Aidroos et al., 2012; Fair et al., 2007; Norman‐Haignere et al., 2012) and low‐pass filtering (Frank, Bowman, et al., 2019; Frank, Preston, et al., 2019; Tambini et al., 2017). Here, we compared connectivity patterns obtained from a rest scan with background connectivity patterns obtained from low‐pass‐filtered task‐based fMRI and FIR residuals obtained from the same task‐based fMRI. In addition to testing the reproduction of large‐scale brain networks, we utilized subject‐specific region of interest (ROI)‐to‐ROI connectivity matrices to evaluate the stability of within‐subject connectivity profiles and individual differences across rest and background connectivity methods.

## 2 METHODS

### 2.1 Participants

Participants were recruited from the University of Oregon and surrounding community for a larger study that included an MRI component for a subset of participants. Only data from the scanned participants are included here and the larger study will not be discussed in the present report. A total of 62 participants were scanned, six of which were excluded: four for falling asleep during the resting‐state scan, one for noncompliance with study procedures, and one for not having enough data following the scrubbing procedures described below. All analyses report the final sample of 56 participants. Participants received written informed consent and were financially compensated for their time. All experimental procedures were approved by Research Compliance Services at the University of Oregon. Participants were eligible for the MRI if they were right‐handed, were native English speakers, had no MRI contraindications, had no psychiatric or neurological illnesses, and were not taking medications known to affect brain function.

### 2.2 Procedure

#### 2.2.1 Overview

In this study, participants underwent fMRI while completing a resting‐state scan and a passive viewing task. During the resting‐state scan (8 min), participants were instructed to keep their eyes open while a fixation cross was projected onto a screen that was viewed through a mirror. Participants then completed four runs (3.67 min each) of task‐based fMRI that consisted of passive viewing of face stimuli shown one at a time every 12 s (2 s stimulus, 10 s fixation intertrial interval). Each run started with a 4‐s fixation cross, followed by a total of 18 trials (nine unique faces, each repeated twice). Participants were instructed to not make any responses during this time. In between the second and third passive viewing run, participants completed an unscanned category learning task where they learned to sort the faces into three families. The results from task‐based fMRI analyses and the categorization task will be reported separately and are not included in the present report. Rather, here we utilize the resting‐state and task‐based fMRI data to address the methodological question of obtaining resting‐state‐like connectivity measures from task‐based designs.

#### 2.2.2 fMRI data acquisition

Scanning was completed on a 3T Siemens Skyra at the UO Lewis Center for Neuroimaging using a 32‐channel head coil. Foam padding was used around the head to minimize motion. The scanning session consisted of a localizer SCOUT sequence, an 8‐min resting‐state scan, four functional 3.67‐min runs of the passive viewing task, and two anatomical scans. Functional data were acquired using a multiband gradient‐echo pulse sequence (repetition time [TR], 2000 ms; echo time [TE], 25 ms; flip angle, 90°; matrix size, 104 × 104; 72 contiguous slices oriented 15° off the anterior commissure–posterior commissure line to reduce prefrontal signal dropout; interleaved acquisition; field of view [FOV], 208 mm; voxel size, 2.0 × 2.0 × 2.0 mm; GRAPPA factor, 2; multiband acceleration factor, 3). Anatomical data were collected using a standard high‐resolution T1‐weighted magnetization‐prepared rapid acquisition gradient echo (MPRAGE) anatomical image (TR, 2500 ms; TE, 3.43 ms; inversion time, 1100 ms; flip angle, 7°; matrix size, 256 × 256; 176 contiguous sagittal slices; FOV, 256 mm; slice thickness, 1 mm; voxel size, 1.0 × 1.0 × 1.0 mm; GRAPPA factor, 2) and a custom anatomical T2 coronal image (TR, 13,520 ms; TE, 88 ms; flip angle, 150°; matrix size, 512 × 512; 65 contiguous slices oriented perpendicularly to the main axis of the hippocampus; interleaved acquisition; FOV, 220 mm; voxel size, 0.4 × 0.4 × 2.0 mm; GRAPPA factor, 2).

#### 2.2.3 fMRI analysis strategy

**FIGURE 1:**
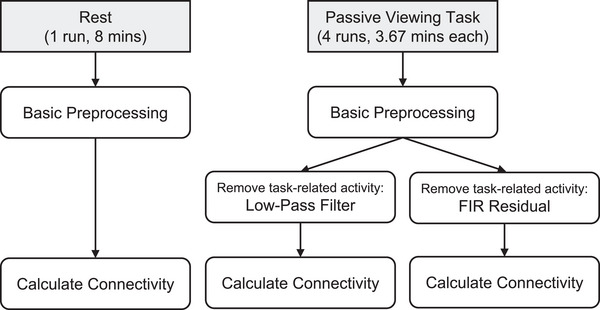
Overview of preprocessing and analysis pipeline. Resting‐state and passive viewing data were subjected to the same preprocessing steps (see Section 2.2.4). ROI‐to‐ROI connectivity was measured from the preprocessed resting‐state data, while data from the passive viewing task were subjected to an additional step of preprocessing. Task‐related activity was removed from the passive viewing data using the low‐pass filter (LPF) and FIR residuals (FIR) approaches. Connectivity was calculated from the LPF data and from the FIR data, resulting in two sets of task‐based connections that were then compared to the “gold standard” rest connectivity.

Here, we aimed to compare two methods for removing task‐related activity, low‐pass filter and FIR residuals, in a slow event‐related fMRI design. Each of these methods were applied to the functional data collected during the passive viewing task to remove task‐evoked activity. Background connectivity was then measured and averaged across the four runs. Functional connectivity was also calculated from the resting‐state scan and used as a “gold standard” to which background connectivity was compared. An overview of the preprocessing and analysis pipeline is shown in Figure 1 and a detailed description of the steps is outlined below.

#### 2.2.4 fMRI preprocessing

Dicom files were converted to nifti format using the “dcm2nii” function from MRIcron (https://www.nitrc.org/projects/mricron) and organized in BIDS format. fMRI preprocessing and data analysis were carried out using FEAT (fMRI Expert Analysis Tool), version 6.00, part of FSL (www.fmrib.ox.ac.uk/fsl), and custom scripts. All functional runs were brain extracted using BET and motion corrected within each run using McFlirt. The functional and MPRAGE anatomical scans of each subject were coregistered to their first functional volume by rigid/affine transformations using the Advanced Neuroimaging Tools (http://stnava.github.io/ANTs/). Noise components from the co‐registered functionals were identified and removed using Independent Component Analysis‐based Automatic Removal of Motion Artifacts (ICA‐AROMA) (https://github.com/maartenmennes/ICA‐AROMA). A high‐pass temporal filter (100 s) was then applied to the denoised functionals. The resulting time series were then ready for the computation of connectivity measures (resting state fMRI) or for the removal of task‐evoked activity (task‐based fMRI).

#### 2.2.5 Removing task‐evoked activity from task‐based fMRI

Functional time series from task‐based fMRI underwent an additional processing step—removal of task‐evoked activity—prior to calculating connectivity measures. Here, we utilized and compared two previously used methods proposed to remove task‐evoked activity, low‐pass filter (Frank, Bowman, et al., 2019; Frank, Preston, et al., 2019; Tambini et al., 2017) and FIR residuals (Al‐Aidroos et al., 2012; Fair et al., 2007; Norman‐Haignere et al., 2012). In the low‐pass filtering method, we applied a low‐pass filter with a 16 s cutoff to remove frequencies in the BOLD signal that were at or higher than the 12 s task frequency. A conservative threshold of 16 s was used to ensure all task‐related activity was removed. The filtered functional time series were then utilized for further connectivity analyses. We will refer to this low‐pass‐filtered task‐based dataset as “LPF.”

For the FIR residuals method, we ran a GLM, modeling task‐evoked BOLD signal using an FIR function rather than canonical hemodynamic response function to maximize model fits. The model included six FIR basis functions to estimate activity at each 2‐s TR time point within the 12‐s trial window (Glover, 1999; Kay et al., 2008). The model also included the nuisance regressors outlined by Power et al. (2012), including the six motion parameters, cerebrospinal fluid signal, white matter signal, whole brain signal, and each of their derivatives. After regressing out the task‐evoked activation, we obtained the residual time series (activation not accounted for by the task) to be utilized for further connectivity analyses. We will refer to this residual timeseries dataset as “FIR.” Thus, from the single original task‐based fMRI dataset, we generated two datasets for measuring background connectivity, one using LPF and one using the FIR residual method to account for task‐evoked activity.

#### 2.2.6 Measuring functional connectivity

As we were interested in the stability of overall connectivity patterns across task and rest, we focus on whole‐brain ROI‐to‐ROI connectivity. To assess the potential of each method to reproduce established functional brain networks, we utilized a brain atlas that contains information regarding network membership for each segmented brain region. We adopted the Schaefer et al. (2018) parcellation scheme, containing 100 parcels organized into seven cortical networks (Yeo et al., 2011). This allowed us to compare 100 × 100 matrices of ROI‐to‐ROI connections (symmetrical along the diagonal) as well as summarize those connections at the network level.

The previous steps provided us with three time‐series datasets: resting‐state fMRI (rest), low‐pass‐filtered data from task‐based fMRI (LPF), and FIR residuals from task‐based fMRI (FIR). The same procedures for measuring connectivity were applied to each of the three datasets. Time series were first extracted from each of the 100 parcels. Volumes that exceeded framewise displacement (FD) > 0.5 mm or DVARS > 0.5% were “scrubbed” or removed from the time series (Power et al., 2012). Pairwise partial correlations were conducted between the scrubbed time series of the 100 parcels controlling for nuisance regressors, including the six motion parameters, cerebrospinal fluid signal, white matter signal, whole brain signal, and each of their derivatives (Power et al., 2012). Note that although the nuisance regressors were already included in the FIR models used to obtain the residual time series, they were also partialled out when calculating connectivity to match the procedures with the rest and LPF datasets. The results did not change when using FIR residual background connectivity measured without the nuisance regressors. Background connectivity estimates for the task‐based (LPF and FIR residual) datasets were calculated separately within each run and then averaged across runs. Three individual runs of the passive viewing task were excluded for program errors (one participant), participant falling asleep (one participant), and noncompliance with study protocols (one participant). The connectivity values for these subjects thus reflect the average of three rather than four runs.

The above procedures generated three 100 × 100 ROI‐to‐ROI correlation matrices for each subject: a rest‐based connectivity matrix (rest), a background connectivity matrix obtained after low‐pass filtering (LPF), and a background connectivity matrix obtained from FIR residuals (FIR). These matrices were then utilized to compare rest and background connectivity in terms of (1) within‐subject connectivity profiles, (2) potential to reproduce known network structures, and (3) individual differences in connectivity strength. Please note that the correlation coefficients denoting connectivity strength are reported raw in text and figures for intuitive reading. The connectivity scores and correlations obtained from the analyses presented below were always Fisher *z* transformed before being used for further statistical analyses, per standard recommendations (Dunn & Clark, 1969; Silver & Dunlap, 1987).

### 2.3 Analyses comparing rest and background connectivity

#### 2.3.1 Similarity of subject‐specific connectivity profiles

It has been argued that an individual's connectivity profile at rest contains idiosyncratic features that may serve as their “connectivity fingerprint” (Finn et al., 2015). To evaluate whether task‐based functional connectivity can be similarly utilized, we asked how closely each individual subject's background connectivity matrices (FIR, LPF) matched their rest‐based connectivity matrix. To quantify the similarity for each subject, the upper triangles (not including the diagonal) of the subject‐specific correlation matrices were vectorized and correlated between each method. As there were 100 ROIs in the atlas, there were 4950 unique ROI‐to‐ROI correlation values for each participant and dataset (rest, LPF, FIR) that represented their subject‐specific connectivity patterns. To evaluate how similar such subject‐specific connectivity patterns are between task and rest, Spearman ranked correlations were conducted between rest connectivity patterns and patterns obtained from each of the background connectivity measures (rest × LPF and rest × FIR). The two background connectivity measures were also correlated with each other (LPF × FIR) to evaluate how similar the subject‐specific estimates were between the two approaches to removing task‐evoked signals when applied to the same original task‐based dataset. To evaluate whether one task‐based method (LPF or FIR) consistently produces patterns more similar to those obtained from rest, we compared the subject‐specific rest × LPF similarity scores with rest × FIR similarity scores, using a paired‐samples *t*‐test.

#### 2.3.2 Reproducing network structure of the brain

We next asked how well each of the background connectivity methods reproduce the seven predefined resting‐state cortical networks as implemented in the seven‐network version of the Schaefer atlas (Schaefer et al., 2018; Yeo et al., 2011). For each participant, we calculated the average within‐network and between‐network connectivity produced by each method (rest, LPF, FIR). Within‐network connectivity was calculated by averaging the estimates for all unique within‐network ROI‐to‐ROI connections. Between‐network connectivity was calculated by averaging all unique between‐network ROI‐to‐ROI connections. We then ran a 3 (method: rest, LPF, FIR) × 2 (connection type: within‐network, between‐network) repeated‐measures ANOVA to compare how defined functional networks were in each dataset. We expected within‐network connectivity to be greater than between‐network connectivity, consistent with the cortical network labels assigned to each parcel by Schaefer and colleagues (2018). Of main interest, however, was the interaction between method and the type of connection (within‐network or between‐network) to compare whether the network structure was more or less pronounced in any dataset.

#### 2.3.3 Stability of individual differences in connectivity

As the last analysis, we compared the stability of individual differences in connection strengths between rest and the background connectivity methods. In other words, we wondered whether individual differences (e.g., some subjects having a particularly strong connection between two regions) identified in rest also consistently appear in the background connectivity estimates. We addressed this question on the level of ROI‐to‐ROI connections and on the level of networks. For each ROI‐to‐ROI connection, we calculated the across‐subject Spearman ranked correlations in the connectivity estimates between rest and LPF, rest and FIR, and LPF and FIR. For example, we took the ROI1–ROI2 connectivity estimates for all subjects from the rest data and correlated them with subjects’ ROI1–ROI2 connectivity estimates from the LPF data. To examine individual differences in large‐scale networks, we additionally collapsed the 100 × 100 ROI‐to‐ROI connectivity estimates (excluding connections on the diagonal) from each subject to a 7 × 7 network‐to‐network matrix. For each unique network‐to‐network connection, we conducted across‐subject Spearman ranked correlations in the connectivity estimates between rest and LPF, rest and FIR, and LPF and FIR. In addition to assessing the overall similarity of rest and background connectivity‐based estimates of individual differences, we also tested whether individual differences from rest connectivity are more consistent with individual differences in the LPF‐based or FIR‐based connectivity measures. A paired‐samples *t*‐test was conducted to compare the rest × LPF correlations to the rest × FIR correlations across all connections.

## 3 RESULTS

### 3.1 Similarity of subject‐specific connectivity profiles across task and rest

**FIGURE 2:**
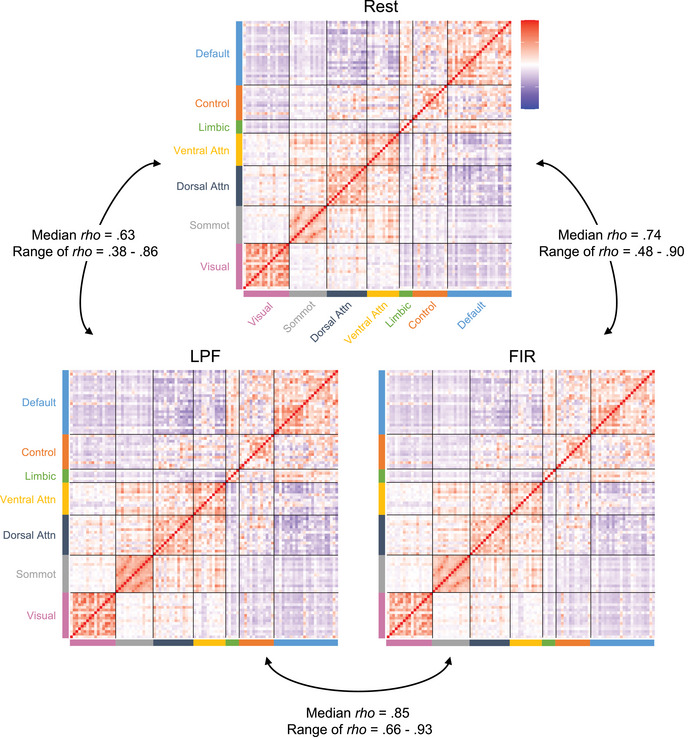
Group‐averaged ROI‐to‐ROI correlation matrices for each method. The ROI‐to‐ROI correlation matrices are shown for the resting‐state data, task‐based LPF data, and the task‐based FIR data. The selected ROIs are from the Schaefer et al. (2018) parcellation scheme and are organized into seven cortical networks (Default, Frontoparietal Control, Limbic, Ventral Attention, Dorsal Attention, Somatomotor, and Visual; Yeo et al., 2011). The background connectivity matrices displayed are averaged across the four runs of passive viewing. For ease of interpretation, the matrices display the raw correlations prior to Fisher *z* transformation. For each subject, pairwise correlations were conducted between the three matrices to determine how well each background connectivity method reproduces the given individual's pattern of connections found during rest. The median and range of these within‐subject correlations are displayed.

**FIGURE 3:**
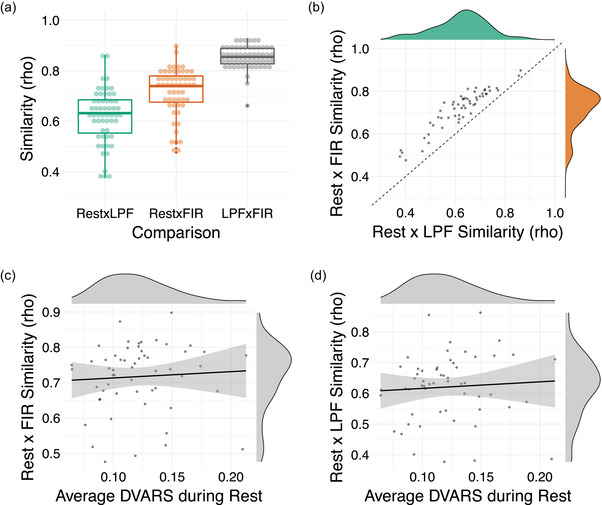
Similarity in individual patterns of connectivity. For each subject, pairwise correlations were conducted to assess the similarity between the individual patterns of connections produced by each method.(A) The distribution of within‐subject similarity scores for each comparison. Each dot represents a participant. The box represents the interquartile range (Q1–Q3), the middle bar represents median (Q2), and the whiskers represent the minimum and maximum values. Points outside of the whisker (>1.5 × interquartile range) are defined as outliers. All pairwise comparisons are statistically significant at *p* < .001 (rest–LPF < rest–FIR < LPF–FIR). (B) A scatter plot showing a strong correlation between rest–LPF similarity scores and rest–FIR similarity scores (*r* = .93, *p* < .001). Those who showed low similarity between rest and task connectivity patterns did so irrespective of the background connectivity method (LPF vs. FIR residuals). Note that all subjects showed higher rest–FIR similarity than rest–LPF similarity (shown by all dots above the line *x* = *y*). (C, D) Scatter plots showing the correlation between average DVARS during rest and subject‐specific similarity scores between rest and task (C: rest × FIR similarity, D: rest × LPF similarity). The correlations were numerically positive but not statistically significant. Thus, low similarity in connectivity estimates between rest and task in some subjects was not clearly attributable to motion.

We first tested how similar subject‐specific patterns of ROI‐to‐ROI connectivity were between rest and each of the background connectivity methods. For each participant, we correlated their ROI‐to‐ROI connectivity pattern from rest with the background connectivity measures from the LPF dataset (rest × LPF) and from the FIR dataset (rest × FIR). The connectivity matrices, averaged across participants, are presented in Figure 2. The subject‐specific background connectivity patterns were moderately to highly correlated with the same participant's connectivity patterns estimated from rest (median rest × LPF pattern similarity rho = .63; median rest × FIR pattern similarity rho = .74). Rest connectivity patterns were more closely matched by background connectivity patterns derived using the FIR method (mean rho(*z*) = .93, *SD* = .19) than LPF method (mean rho(*z*) = .75, *SD* = .18; *t*(55) = −18.95, *p* < .001, *η*_g_^2^ = .87; Figure 3A). Nevertheless, patterns of background connectivity from LPF and FIR residuals data were highly correlated with each other (median rho = .85) and thus both methods likely produce very similar results in practice.

Although the subject‐specific patterns of connectivity were typically consistent between task and rest, some subjects exhibited consistency as low as rho near .4 (see ranges presented in Figure 2 and the distributions of similarity scores in Figure 3A). We were interested if those who demonstrate low rest–LPF similarity also show low rest–FIR similarity. Indeed, the pattern similarity scores were highly correlated (*r* = .93, *p* < .001; Figure 3B). This indicates that low consistency scores were not driven by a specific method of calculating background connectivity but instead were found for a given individual with either method.

We reasoned that perhaps lower similarity may be seen for participants who moved more during scanning and whose connectivity estimates may thus be less reliable. To test this idea, we correlated the rest × task pattern similarity scores with four indices of individual differences in motion: average DVARS, average FD, max DVARS, and max FD. The results did *not* show a clear relationship to motion. For rest × FIR similarity scores, the only numerical relationship we found was with the average DVARS during the rest scan, such that subjects who had higher rest × task similarity tended to have higher average DVARS (rho(54) = .19, *p* = .166) (Figure 3C). The rest × LPF similarity scores showed a similar relationship (rho(54) = .18, *p* = .182). However, the relationships were relatively weak and no correlations with motion reached significance (all uncorrected *p* > .05). Thus, while motion may play a role in the consistency of connectivity patterns between task and rest, our data do not allow us to clearly attribute lower task–rest consistency in some subjects to motion.

### 3.2 Reproducing network structure of the brain

**FIGURE 4:**
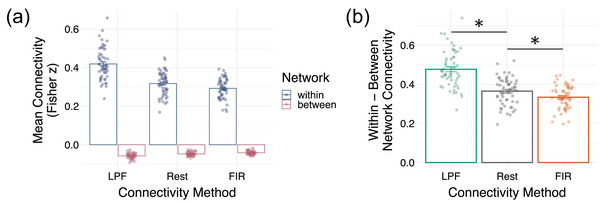
Reproducing resting‐state network structure. (A) Mean within‐network and between‐network connectivity estimates for each method are plotted with data points for individual subjects. Error bars denote ±1 *SE*. (B) The mean within‐network minus between‐network connectivity differences, plotted for each method. Data points for individual subjects are included. Error bars denote ±1 *SE*.

Next, we asked how well each of the background connectivity methods reproduces large‐scale brain networks (Schaefer et al., 2018; Yeo et al., 2011), using the network definitions from the Schaefer et al. (2018) parcellation atlas. A visual inspection of Figure 2 indicates that network structure was clearly observable in all three datasets (rest, LPF, FIR). To formally quantify network organization, all unique connections from the 100 × 100 ROI‐to‐ROI connection matrices (excluding the diagonal) were divided into within‐network connections and between‐network connections, separately for each subject. The average within‐network and average between‐network connection values from each subject and dataset were then submitted to a 3 (rest, LPF, FIR) × 2 (within‐network, between‐network) repeated‐measures ANOVA to test whether the strength of network organization varies among datasets (Figure 4).

As expected, average connectivity within networks (mean rho(*z*) = .34, *SD* = .09) was significantly higher than between networks (mean rho(*z*) = −.05, *SD* = .01; *F*(1,55) = 2125.79, *p* < .001, *η*_g_^2^ = .95), confirming that the network organization of the Schaefer et al. (2018) parcellation was reflected in our data. We also found a main effect of connectivity method (*F*(1.41, 77.6) = 135.13, *p* < .001, *η*_g_^2^ = .21, Greenhouse Geiser corrected). Follow‐up comparisons revealed the LPF method (mean rho(*z*) = .18, *SD* = .25) produced significantly higher connections compared to rest (mean rho(*z*) = .135, *SD* = .19; *t*(55) = 9.97, *p* < .001, *η*_g_^2^ = .36) and FIR methods (mean rho(*z*) = .125, *SD* = .17; *t*(55) = 19.24, *p* < .001, *η*_g_^2^ = .48), while the FIR method produced the smallest average connectivity values (comparison to rest: *t*(55) = 3.02, *p* = .004, *η*_g_^2^ = .04).

Of main interest, however, was the interaction between the connectivity method (rest, LPF, FIR) and the type of connection (within‐network, between‐network). We found a significant interaction indicating that the network structure (the difference between within‐network and between‐network connectivity) varied among datasets (*F*(1.41, 77.6) = 123.29, *p* < .001, *η*_g_^2^ = .30, Greenhouse Geiser corrected; Figure 4). Interestingly, network structure was most pronounced in the LPF‐derived background connectivity estimates, followed by rest, followed by FIR residual‐derived background connectivity estimates (Figure 4B). Follow‐up pairwise *t*‐tests were all statistically significant: LPF compared to rest (*t*(55) = 9.01, *p* < .001, *η*_g_^2^ = .32), rest versus FIR (*t*(55) = 3.86, *p* < .001, *η*_g_^2^ = .05), and LPF versus FIR (*t*(55) = 18.65, *p* < .001, *η*_g_^2^ = .46). The smallest difference was between rest and the FIR background connectivity method rather than between the two background connectivity methods, with FIR method producing somewhat less pronounced network structure (see also Figure 2 for the full ROI‐to‐ROI matrices further demonstrating this effect). Nevertheless, network structure was clearly reflected in all three datasets, with significantly higher within‐network than between‐network connectivity in every dataset and every individual subject (Figure 4).

### 3.3 Stability of individual differences in connectivity across task and rest

**FIGURE 5:**
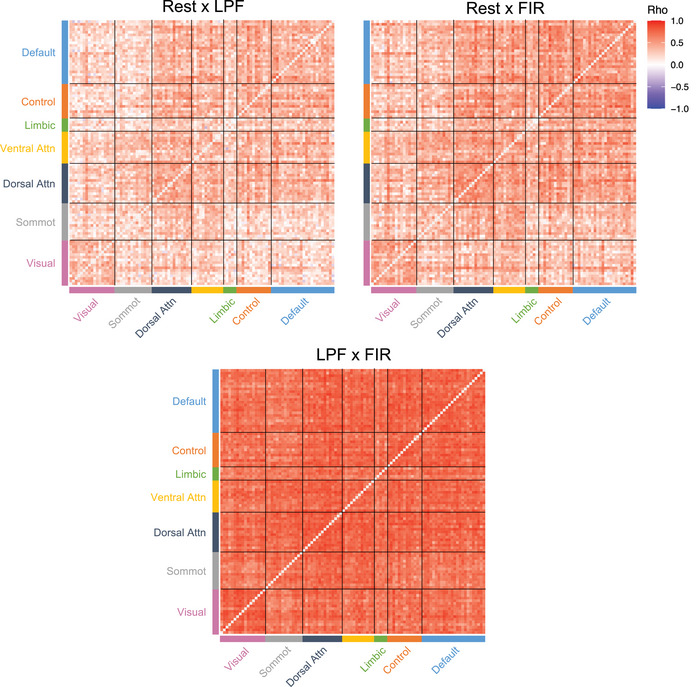
Stability of individual differences for each ROI‐to‐ROI connection. For each unique ROI‐to‐ROI connection, individual differences in connectivity estimates were correlated between rest and LPF, rest and FIR, and LPF and FIR datasets.

**FIGURE 6:**
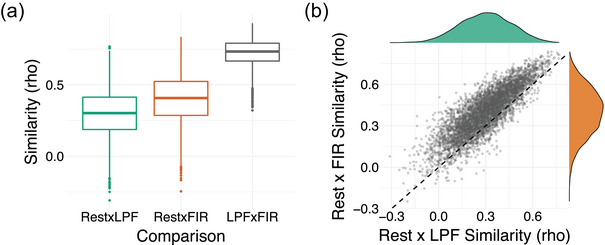
Stability of individual differences between the methods. For each ROI‐to‐ROI connection, individual differences in connectivity were calculated between two methods (rest × LPF, rest × FIR, and LPF × FIR). (A) The boxplot of similarity scores (Spearman rho) for all unique ROI‐to‐ROI connections. The box represents the interquartile range (Q1–Q3), the middle bar represents median (Q2), and the whiskers represent the minimum and maximum values. All pairwise comparisons are statistically significant at *p* < .001 (rest–LPF < rest–FIR < LPF–FIR). (B) Scatterplot showing a strong correlation between rest–LPF similarity and rest–FIR similarity (*r* = .85, *p* < .001). The connections that showed low similarity between rest and FIR also showed low similarity between rest and LPF, suggesting that some connections showed relatively low stability of individual differences between rest and task that was not driven by a particular method for removing task‐related activity. Dashed line indicates where *x* = *y*.

Prior work suggested that individual differences in resting‐state connectivity are meaningful, related to various individual characteristics, such as personality, cognition, or clinical symptoms (Finn et al., 2015; Liu et al., 2019; Reinen et al., 2018; Rosenberg et al., 2015; Takamura & Hanakawa, 2017; Toschi et al., 2018). Here, we asked whether individual differences in background connectivity are similar to those observed at rest. First, we examined the stability of individual differences on the level of ROI‐to‐ROI connections. For each ROI‐to‐ROI connection, across‐subject correlations were conducted between rest and LPF, rest and FIR, and LPF and FIR. For example, subject estimates in ROI1–ROI2 connectivity during rest were correlated with subject estimates in ROI1–ROI2 connectivity derived from the LPF method. This resulted in an estimate of stability for each of the 4950 ROI‐to‐ROI connections for each of the three comparisons (rest × LPF, rest × FIR, and LPF × FIR). The between‐datasets similarity of individual differences estimates for each connection is visualized in Figure 5 and summarized in Table 1 and Figure 6.

**TABLE 1:** Summary of correlations in individual differences for ROI‐to‐ROI connections

Comparison	Mean rho	Median rho	*SD* rho	Range
Rest × LPF	.30	.30	.16	−.31 to .77
Rest × FIR	.40	.41	.17	−.25 to .84
LPF × FIR	.72	.73	.09	.32 to .93

On average, individual differences in background connectivity were weakly to moderately correlated with individual differences in resting‐state connectivity, suggesting that individual differences in specific ROI‐to‐ROI connections are not always stable from rest to task (Table 1; Figure 6A). Individual differences in background connectivity obtained from the FIR method were more correlated with rest than those obtained from the LPF method (*t*(4949) = 78.22, *p* < .001, *η*_g_^2^ = .09).

Individual differences for some connections showed especially low similarity between rest and task (lowest rho = −.31). To determine whether rest–task differences were common for certain connections or whether they were driven by a particular background connectivity method, we correlated rest × LPF similarity scores with rest × FIR similarity scores. We found that rest–FIR similarity was strongly and positively correlated with rest–LPF similarity (rho(4948) = .85, *p* < .001), with connections showing low similarity in individual differences between rest and LPF also showing low similarity between rest and FIR (Figure 6B). This finding suggests that for some regions, individual differences in connection strength can vary considerably between rest and task, irrespective of the method used for removing task‐evoked activity (LPF, FIR).

**FIGURE 7:**
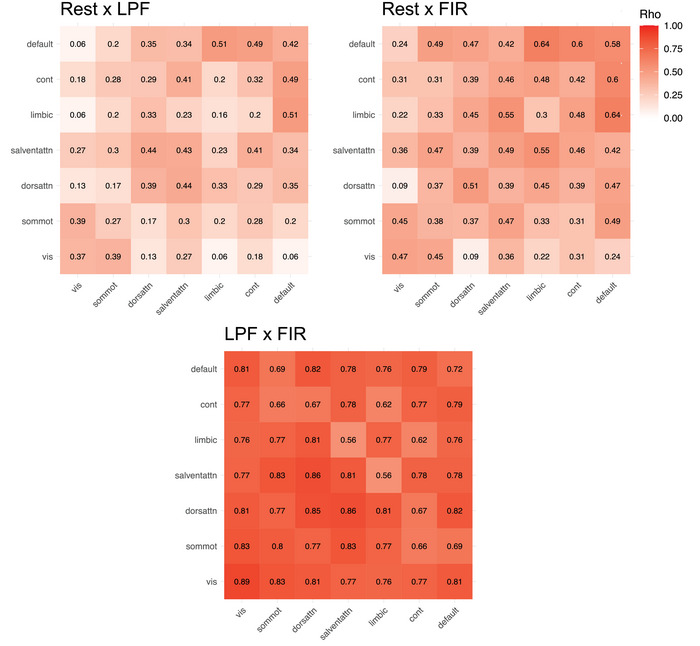
Stability of individual differences in network‐to‐network connections. The 100 × 100 ROI‐to‐ROI connections were collapsed into 7 × 7 network‐to‐network connections. For each network‐to‐network connection, individual differences in connectivity were correlated between two methods (rest × LPF, rest × FIR, LPF × FIR).

Notably, given research on task‐driven changes in connectivity (Cole et al., 2021; Elton & Gao, 2015; Rissman et al., 2004), these differences between task and rest may be meaningful, reflecting differential modulation of regional or network connections in different subjects. We thus wanted to explore the degree of stability across task and rest for different connections more qualitatively. First, we collapsed across the 100 × 100 ROI‐to‐ROI connections to 7 × 7 network‐to‐network connections, reducing the number of connections to consider while respecting known functional organization of the brain. Individual differences in connectivity for each of the unique network‐to‐network connections were then correlated between rest and LPF, rest and FIR, and LPF and FIR. The correlations, visualized in Figure 7 and summarized in Table 2, are of similar average strength as those found on the level of ROI‐to‐ROI connections. Similar to the ROI‐to‐ROI findings, individual differences in network‐to‐network connections demonstrated greater rest–FIR similarity than rest–LPF similarity (*t*(27) = 7.93, *p* < .001, *η*_g_^2^ = .21).

**TABLE 2:** Summary of correlations in individual differences for network‐to‐network connections

Comparison	Mean rho	Median rho	*SD* rho	Range
Rest × LPF	.29	.29	.12	.06–.51
Rest × FIR	.42	.44	.12	.09–.64
LPF × FIR	.77	.78	.07	.56–.89

As can be observed from Figure 7, individual differences in the default mode network connections appeared the most similar between rest and task, both within the default network and between the default network and other networks. Individual differences of visual network connections, especially with the dorsal attention network and perhaps limbic and default networks (for LPF), appeared the least similar. Thus, individual differences in connectivity were not always stable from rest to task, driven by some connections more than others. In contrast, individual differences estimates of background connectivity were fairly consistent across the two analytical methods (LPF, FIR), both at the level of individual connections (Table 1; Figures 5 and 6) and at the level of network connections (Table 2; Figure 7).

## 4 DISCUSSION

Resting‐state connectivity has substantially contributed to our understanding of brain organization and how individual differences in connectivity strengths and patterns are linked to behavioral, clinical, and other individual factors (Ferreira & Busatto, 2013; Fong et al., 2019; Geerligs et al., 2015; Liu et al., 2019; Poole et al., 2016; Reinen et al., 2018; Tracy & Doucet, 2015). Previous research suggests that brain activity occurring in the background of a task can be used to reproduce resting‐state‐like connectivity profiles (Cole et al., 2014; Gratton et al., 2018; Kraus et al., 2021), allowing researchers to get further use out of existing task‐based fMRI datasets. Here, we tested this idea along with two methods for removing task‐related activity in a slow event‐related fMRI task design: low‐pass filter and GLM. Our results demonstrated that background connectivity derived from task‐based fMRI successfully reproduced large‐scale cortical networks found at rest and largely maintained within‐subject patterns of ROI‐to‐ROI connectivity. In contrast, individual differences in connectivity strength were less stable across task and rest for many connections, potentially reflecting task‐driven modulations of connectivity strength that differ across individuals. Across all analyses, rest‐based connectivity measures were more closely approximated by background connectivity derived after general linear modeling than low‐pass filtering of task‐evoked activation. Nevertheless, both methods for removing task‐evoked activity generated connectivity patterns similar to rest and to one another, suggesting both are viable approaches for measuring background connectivity.

A significant contribution of resting‐state connectivity has been the identification of large‐scale networks, which can be leveraged for clinical research (Reinen et al., 2018; Takamura & Hanakawa, 2017; Tracy & Doucet, 2015) and understanding human behavior and cognition (Cohen & D'Esposito, 2016; Shine et al., 2016). Here, we reproduced large‐scale cortical networks during rest (Schaefer et al., 2018; Yeo et al., 2011) and demonstrated that functional networks are also maintained in background connectivity derived from task‐based fMRI. Previous studies have shown similar organization of functional networks between rest and task (Bzdok et al., 2016; Smith et al., 2009), with tasks recruiting the same intrinsic networks derived from rest (Gess et al., 2014; Shah et al., 2016). Together, these findings suggest that large‐scale cortical networks may reflect inherent and stable properties of brain organization.

One inspiration for our study was prior work suggesting that individuals display unique functional connectivity profiles (“connectivity fingerprints”) that can be leveraged to predict individual's clinical, cognitive, and other characteristics (Finn et al., 2015; Fong et al., 2019; Liu et al., 2019; Rosenberg et al., 2015; Takamura & Hanakawa, 2017; Toschi et al., 2018; Tracy & Doucet, 2015). Aligned with the fingerprint hypothesis, we found that idiosyncratic patterns of ROI‐to‐ROI connectivity during task‐based fMRI were highly similar to those found at rest for most participants (see also Cole et al., 2014; Gratton et al., 2018; Kraus et al., 2021). As individual behaviors or traits can be linked to specific connections or networks, rather than a whole‐brain profile (Gerraty et al., 2014; Klumpp et al., 2014; Poole et al., 2016; Qin et al., 2014; Toschi et al., 2018), we also asked whether individual differences for specific connections are maintained between rest‐ and task‐based fMRI. Unexpectedly, individual differences in ROI‐to‐ROI and network‐to‐network connections were only weakly to moderately correlated between rest and task. These differences are unlikely to reflect just noise, given that they were observed even when averaging across many connections for network‐to‐network analyses. Instead, the differences between rest and background connectivity may be driven by engagement of specific connections during the task. Indeed, Fair and colleagues (2007) showed how connections that were unique to the task‐based data overlapped with regions that were also activated during the task. Thus, although background connectivity reflects idiosyncratic profiles stable within an individual, it is also modulated by the task hand, reflecting different brain states (Al‐Aidroos et al., 2012; Cooper & Ritchey, 2019; Tambini et al., 2017).

Our finding that individual differences in connectivity strength are not always consistent between rest and task opens up the question of which measure is more relevant to behavior and other individual characteristics. Prior studies have demonstrated the importance of task‐modulated functional connectivity in predicting behavior (Elton & Gao, 2015; Greene et al., 2018; Jiang et al., 2020), with some suggesting it may even serve as a better predictor compared to rest (Greene et al., 2018; Jiang et al., 2020). Moreover, Gratton and colleagues (2018) found that task modulations of functional networks were largely due to individual variations in connectivity changes between task and rest, which could reflect individual differences in task engagement. Here, we did not have a suitable behavioral measure of individual differences and our sample size would not provide sufficient power to compare brain–behavior relationship between task and rest. However, it would be informative to compare the functional significance of rest‐derived and task‐derived individual differences measures in future studies.

In addition to comparing rest and background connectivity, we compared the low‐pass filtering and GLM methods for removing task‐related activity. When examining individuals, background connectivity derived from the FIR residual dataset demonstrated greater similarity with rest connectivity compared to the LPF dataset. This was true for both idiosyncratic patterns of connectivity and individual differences in the strength of connections. Nevertheless, the connectivity measures derived from the two methods of removing task‐evoked activity were highly correlated with each other and were similarly related to rest, such that any connections or individuals that appeared to more strongly differ across task and rest did so irrespective of a particular method. Therefore, while the FIR residuals method generates measures that more closely match rest‐based measures, the LPF method provides nearly identical results while being computationally simpler and faster. Thus, researchers can choose either method of removing task‐related activity based on their needs, with existing and future studies using one or the other being directly comparable.

The only notable difference between the two background connectivity methods was found in the reproduction of the large‐scale cortical networks, where the LPF method produced networks *more* pronounced than those found during rest, while the FIR method produced networks *less* pronounced than rest. Because the underlying network modularity must be the same between the LPF and FIR datasets, as both are coming from the same raw data, these results indicate that the absolute value of connection strength is differentially affected by various analysis steps (such as signal smoothing) and may not be directly comparable unless identical analysis steps are used.

The current study compared background connectivity obtained after statistically removing task‐evoked activity from a slow event‐related task‐based fMRI with connectivity estimates obtained from resting‐state fMRI in the same set of subjects. Our findings contribute new evidence that background connectivity can capture the intrinsic structure of functional connectivity as found during rest. Specifically, background connectivity measures preserve the organization of large‐scale functional networks, as well as idiosyncratic connectivity patterns within subjects. In contrast, individual differences in the strength of connections vary more substantially between rest and task, opening up the question of which measure may be more relevant to behavior. Our study was also the first to compare two methods for removing task‐evoked activity from slow event‐related fMRI. Background connectivity was most similar to rest when derived from the residuals of a GLM, though connectivity derived from a low‐pass filter produced highly similar results and may have its own advantages. Together, our findings highlight the utility of background connectivity for capturing “connectivity fingerprints” and the viability of the different approaches for removing task‐related activity.

## CONFLICT OF INTEREST STATEMENT

The authors declare no conflicts of interest.

### PEER REVIEW

The peer review history for this article is available at https://publons.com/publon/10.1002/brb3.3015.

## Data Availability

The data for this study are available through OpenNeuro at https://openneuro.org/datasets/ds004349.

## References

[brb33015-bib-0001] Al‐Aidroos, N. , Said, C. P. , & Turk‐Browne, N. B. (2012). Top‐down attention switches coupling between low‐level and high‐level areas of human visual cortex. Proceedings of the National Academy of Sciences of the United States of America, 109(36), 14675–14680. 10.1073/pnas.1202095109 22908274 PMC3437858

[brb33015-bib-0002] Beckmann, C. F. , DeLuca, M. , Devlin, J. T. , & Smith, S. M. (2005). Investigations into resting‐state connectivity using independent component analysis. Philosophical Transactions of the Royal Society B: Biological Sciences, 360(1457), 1001–1013. 10.1098/rstb.2005.1634 PMC1854918 16087444

[brb33015-bib-0003] Bzdok, D. , Varoquaux, G. , Grisel, O. , Eickenberg, M. , Poupon, C. , & Thirion, B. (2016). Formal models of the network co‐occurrence underlying mental operations. PLoS Computational Biology, 12(6), 1–31. 10.1371/journal.pcbi.1004994 PMC4911040 27310288

[brb33015-bib-0004] Clark, C. M. , Kessler, R. , Buchsbaum, M. S. , Margolin, R. A. , & Holcomb, H. H. (1984). Correlational methods for determining regional coupling of cerebral glucose metabolism: A pilot study. Biological Psychiatry, 19(5), 663–678. http://www.ncbi.nlm.nih.gov/pubmed/6610442 6610442

[brb33015-bib-0005] Cohen, J. R. , & D'Esposito, M. (2016). The segregation and integration of distinct brain networks and their relationship to cognition. Journal of Neuroscience, 36(48), 12083–12094. 10.1523/JNEUROSCI.2965-15.2016 27903719 PMC5148214

[brb33015-bib-0006] Cole, M. W. , Bassett, D. S. , Power, J. D. , Braver, T. S. , & Petersen, S. E. (2014). Intrinsic and task‐evoked network architectures of the human brain. Neuron, 83(1), 238–251. 10.1016/j.neuron.2014.05.014 24991964 PMC4082806

[brb33015-bib-0007] Cole, M. W. , Ito, T. , Cocuzza, C. , & Sanchez‐Romero, R. (2021). The functional relevance of task‐state functional connectivity. Journal of Neuroscience, 41(12), 2684–2702. 10.1523/JNEUROSCI.1713-20.2021 33542083 PMC8018740

[brb33015-bib-0008] Cooper, R. A. , & Ritchey, M. (2019). Cortico‐hippocampal network connections support the multidimensional quality of episodic memory. eLife, 8, 1–22. 10.7554/eLife.45591 PMC6450667 30900990

[brb33015-bib-0009] Dosenbach, N. U. F. , Fair, D. A. , Miezin, F. M. , Cohen, A. L. , Wenger, K. K. , Dosenbach, R. A. T. , & Petersen, S. E. (2007). Distinct brain networks for adaptive and stable task control in humans. Proceedings of the National Academy of Sciences of the United States of America, 104(26), 11073–11078. 10.1073/pnas.0704320104 17576922 PMC1904171

[brb33015-bib-0010] Dosenbach, N. U. F. , Nardos, B. , Cohen, A. L. , Fair, D. A. , Power, J. D. , Church, J. A. , & Schlaggar, B. L. (2010). Prediction of individual brain maturity using fMRI. Science, 329(5997), 1358–1361. 10.1126/science.1194144 20829489 PMC3135376

[brb33015-bib-0011] Duncan, K. , Tompary, A. , & Davachi, L. (2014). Associative encoding and retrieval are predicted by functional connectivity in distinct hippocampal area CA1 pathways. Journal of Neuroscience, 34(34), 11188–11198. 10.1523/JNEUROSCI.0521-14.2014 25143600 PMC4138331

[brb33015-bib-0012] Dunn, O. J. , & Clark, V. (1969). Correlation coefficients measured on the same individuals. Journal of the American Statistical Association, 64(325), 366–377. 10.1080/01621459.1969.10500981

[brb33015-bib-0013] Elton, A. , & Gao, W. (2015). Task‐related modulation of functional connectivity variability and its behavioral correlations. Human Brain Mapping, 36(8), 3260–3272. 10.1002/hbm.22847 26015070 PMC6869497

[brb33015-bib-0014] Fair, D. A. , Schlaggar, B. L. , Cohen, A. L. , Miezin, F. M. , Dosenbach, N. U. F. , Wenger, K. K. , & Petersen, S. E. (2007). A method for using blocked and event‐related fMRI data to study “resting state” functional connectivity. Neuroimage, 35(1), 396–405. 10.1016/j.neuroimage.2006.11.051 17239622 PMC2563954

[brb33015-bib-0015] Ferreira, L. K. , & Busatto, G. F. (2013). Resting‐state functional connectivity in normal brain aging. Neuroscience and Biobehavioral Reviews, 37(3), 384–400. 10.1016/j.neubiorev.2013.01.017 23333262

[brb33015-bib-0016] Finn, E. S. , Shen, X. , Scheinost, D. , Rosenberg, M. D. , Huang, J. , Chun, M. M. , & Constable, R. T. (2015). Functional connectome fingerprinting: Identifying individuals using patterns of brain connectivity. Nature Neuroscience, 18(11), 1664–1671. 10.1038/nn.4135 26457551 PMC5008686

[brb33015-bib-0017] Fong, A. H. C. , Yoo, K. , Rosenberg, M. D. , Zhang, S. , Li, C. S. R. , Scheinost, D. , & Chun, M. M. (2019). Dynamic functional connectivity during task performance and rest predicts individual differences in attention across studies. Neuroimage, 188, 14–25. 10.1016/j.neuroimage.2018.11.057 30521950 PMC6401236

[brb33015-bib-0018] Fox, M. D. , Corbetta, M. , Snyder, A. Z. , Vincent, J. L. , & Raichle, M. E. (2006). Spontaneous neuronal activity distinguishes human dorsal and ventral attention systems. Proceedings of the National Academy of Sciences of the United States of America, 103(26), 10046–10051. 10.1073/pnas.0604187103 16788060 PMC1480402

[brb33015-bib-0019] Fox, M. D. , & Raichle, M. E. (2007). Spontaneous fluctuations in brain activity observed with functional magnetic resonance imaging. Nature Reviews Neuroscience, 8(9), 700–711. 10.1038/nrn2201 17704812

[brb33015-bib-0020] Fox, M. D. , Snyder, A. Z. , Zacks, J. M. , & Raichle, M. E. (2006). Coherent spontaneous activity accounts for trial‐to‐trial variability in human evoked brain responses. Nature Neuroscience, 9(1), 23–25. 10.1038/nn1616 16341210

[brb33015-bib-0021] Frank, L. E. , Bowman, C. R. , & Zeithamova, D. (2019). Differential functional connectivity along the long axis of the hippocampus aligns with differential role in memory specificity and generalization. Journal of Cognitive Neuroscience, 31(12), 1958–1975. 10.1162/jocn_a_01457 31397613 PMC8080992

[brb33015-bib-0022] Frank, L. E. , Preston, A. R. , & Zeithamova, D. (2019). Functional connectivity between memory and reward centers across task and rest track memory sensitivity to reward. Cognitive, Affective and Behavioral Neuroscience, 19(3), 503–522. 10.3758/s13415-019-00700-8 PMC7137582 30805850

[brb33015-bib-0023] Friston, K. J. (1994). Functional and effective connectivity in neuroimaging: A synthesis. Human Brain Mapping, 2(1–2), 56–78. 10.1002/hbm.460020107

[brb33015-bib-0024] Friston, K. J. , Frith, C. D. , Liddle, P. F. , & Frackowiak, R. S. J. (1993). Functional connectivity: The principal‐component analysis of large (PET) data sets. Journal of Cerebral Blood Flow & Metabolism, 13(1), 5–14. 10.1038/jcbfm.1993.4 8417010

[brb33015-bib-0025] Geerligs, L. , Renken, R. J. , Saliasi, E. , Maurits, N. M. , & Lorist, M. M. (2015). A brain‐wide study of age‐related changes in functional connectivity. Cerebral Cortex, 25(7), 1987–1999. 10.1093/cercor/bhu012 24532319

[brb33015-bib-0026] Gerraty, R. T. , Davidow, J. Y. , Wimmer, G. E. , Kahn, I. , & Shohamy, D. (2014). Transfer of learning relates to intrinsic connectivity between hippocampus, ventromedial prefrontal cortex, and large‐scale networks. Journal of Neuroscience, 34(34), 11297–11303. 10.1523/JNEUROSCI.0185-14.2014 25143610 PMC4138340

[brb33015-bib-0027] Gess, J. L. , Fausett, J. S. , Kearney‐Ramos, T. E. , Kilts, C. D. , & James, G. A. (2014). Task‐dependent recruitment of intrinsic brain networks reflects normative variance in cognition. Brain and Behavior, 4(5), 650–664. 10.1002/brb3.243 25328842 PMC4107383

[brb33015-bib-0028] Glover, G. H. (1999). Deconvolution of impulse response in event‐related BOLD fMRI. Neuroimage, 9(4), 416–429. 10.1006/nimg.1998.0419 10191170

[brb33015-bib-0029] Gratton, C. , Laumann, T. O. , Nielsen, A. N. , Greene, D. J. , Gordon, E. M. , Gilmore, A. W. , & Petersen, S. E. (2018). Functional brain networks are dominated by stable group and individual factors, not cognitive or daily variation. Neuron, 98(2), 439–452.e5. 10.1016/j.neuron.2018.03.035 29673485 PMC5912345

[brb33015-bib-0030] Greene, A. S. , Gao, S. , Scheinost, D. , & Constable, R. T. (2018). Task‐induced brain state manipulation improves prediction of individual traits. Nature Communications, 9(1), 2807. 10.1038/s41467-018-04920-3 PMC6052101 30022026

[brb33015-bib-0031] Greicius, M. D. , Krasnow, B. , Reiss, A. L. , & Menon, V. (2003). Functional connectivity in the resting brain: A network analysis of the default mode hypothesis. Proceedings of the National Academy of Sciences of the United States of America, 100(1), 253–258. 10.1073/pnas.0135058100 12506194 PMC140943

[brb33015-bib-0032] Greicius, M. D. , Supekar, K. , Menon, V. , & Dougherty, R. F. (2009). Resting‐state functional connectivity reflects structural connectivity in the default mode network. Cerebral Cortex, 19(1), 72–78. 10.1093/cercor/bhn059 18403396 PMC2605172

[brb33015-bib-0033] Guo, C. C. , Kurth, F. , Zhou, J. , Mayer, E. A. , Eickhoff, S. B. , Kramer, J. H. , & Seeley, W. W. (2012). One‐year test‐retest reliability of intrinsic connectivity network fMRI in older adults. Neuroimage, 61(4), 1471–1483. 10.1016/j.neuroimage.2012.03.027 22446491 PMC4226138

[brb33015-bib-0034] Honey, C. J. , Kötter, R. , Breakspear, M. , & Sporns, O. (2007). Network structure of cerebral cortex shapes functional connectivity on multiple time scales. Proceedings of the National Academy of Sciences, 104(24), 10240–10245. 10.1073/pnas.0701519104 PMC1891224 17548818

[brb33015-bib-0035] Horien, C. , Shen, X. , Scheinost, D. , & Constable, R. T. (2019). The individual functional connectome is unique and stable over months to years. Neuroimage, 189, 676–687. 10.1016/j.neuroimage.2019.02.002 30721751 PMC6422733

[brb33015-bib-0036] Jiang, R. , Zuo, N. , Ford, J. M. , Qi, S. , Zhi, D. , Zhuo, C. , & Sui, J. (2020). Task‐induced brain connectivity promotes the detection of individual differences in brain‐behavior relationships. Neuroimage, 207, 116370. 10.1016/j.neuroimage.2019.116370 31751666 PMC7345498

[brb33015-bib-0037] Kay, K. N. , David, S. V. , Prenger, R. J. , Hansen, K. A. , & Gallant, J. L. (2008). Modeling low‐frequency fluctuation and hemodynamic response timecourse in event‐related fMRI. Human Brain Mapping, 29(2), 142–156. 10.1002/hbm.20379 17394212 PMC6871156

[brb33015-bib-0038] Klumpp, H. , Keutmann, M. K. , Fitzgerald, D. A. , Shankman, S. A. , & Phan, K. L. (2014). Resting state amygdala‐prefrontal connectivity predicts symptom change after cognitive behavioral therapy in generalized social anxiety disorder. Biology of Mood & Anxiety Disorders, 4(1), 14. 10.1186/s13587-014-0014-5 25540682 PMC4276016

[brb33015-bib-0039] Kraus, B. T. , Perez, D. , Ladwig, Z. , Seitzman, B. A. , Dworetsky, A. , Petersen, S. E. , & Gratton, C. (2021). Network variants are similar between task and rest states. Neuroimage, 229(2020), 117743. 10.1016/j.neuroimage.2021.117743 33454409 PMC8080895

[brb33015-bib-0040] Liu, W. , Kohn, N. , & Fernández, G. (2019). Intersubject similarity of personality is associated with intersubject similarity of brain connectivity patterns. Neuroimage, 186, 56–69. 10.1016/j.neuroimage.2018.10.062 30389630

[brb33015-bib-0041] Norman‐Haignere, S. V. , McCarthy, G. , Chun, M. M. , & Turk‐Browne, N. B. (2012). Category‐selective background connectivity in ventral visual cortex. Cerebral Cortex, 22(2), 391–402. 10.1093/cercor/bhr118 21670097 PMC3256407

[brb33015-bib-0042] Passingham, R. E. , Stephan, K. E. , & Kötter, R. (2002). The anatomical basis of functional localization in the cortex. Nature Reviews Neuroscience, 3(8), 606–616. 10.1038/nrn893 12154362

[brb33015-bib-0043] Poole, V. N. , Robinson, M. E. , Singleton, O. , DeGutis, J. , Milberg, W. P. , McGlinchey, R. E. , & Esterman, M. (2016). Intrinsic functional connectivity predicts individual differences in distractibility. Neuropsychologia, 86, 176–182. 10.1016/j.neuropsychologia.2016.04.023 27132070

[brb33015-bib-0044] Power, J. D. , Barnes, K. A. , Snyder, A. Z. , Schlaggar, B. L. , & Petersen, S. E. (2012). Spurious but systematic correlations in functional connectivity MRI networks arise from subject motion. Neuroimage, 59(3), 2142–2154. 10.1016/j.neuroimage.2011.10.018 22019881 PMC3254728

[brb33015-bib-0045] Qin, S. , Young, C. B. , Duan, X. , Chen, T. , Supekar, K. , & Menon, V. (2014). Amygdala subregional structure and intrinsic functional connectivity predicts individual differences in anxiety during early childhood. Biological Psychiatry, 75(11), 892–900. 10.1016/j.biopsych.2013.10.006 24268662 PMC3984386

[brb33015-bib-0046] Reinen, J. M. , Chén, O. Y. , Hutchison, R. M. , Yeo, B. T. T. , Anderson, K. M. , Sabuncu, M. R. , & Holmes, A. J. (2018). The human cortex possesses a reconfigurable dynamic network architecture that is disrupted in psychosis. Nature Communications, 9(1), 1–15. 10.1038/s41467-018-03462-y PMC5861099 29559638

[brb33015-bib-0047] Rissman, J. , Gazzaley, A. , & D'Esposito, M. (2004). Measuring functional connectivity during distinct stages of a cognitive task. Neuroimage, 23(2), 752–763. 10.1016/j.neuroimage.2004.06.035 15488425

[brb33015-bib-0048] Rosenberg, M. D. , Finn, E. S. , Scheinost, D. , Papademetris, X. , Shen, X. , Constable, R. T. , & Chun, M. M. (2015). A neuromarker of sustained attention from whole‐brain functional connectivity. Nature Neuroscience, 19(1), 165–171. 10.1038/nn.4179 26595653 PMC4696892

[brb33015-bib-0049] Rykhlevskaia, E. , Gratton, G. , & Fabiani, M. (2008). Combining structural and functional neuroimaging data for studying brain connectivity: A review. Psychophysiology, 45(2), 173–187. 10.1111/j.1469-8986.2007.00621.x 17995910

[brb33015-bib-0050] Schaefer, A. , Kong, R. , Gordon, E. M. , Laumann, T. O. , Zuo, X.‐N. , Holmes, A. J. , & Yeo, B. T. T. (2018). Local‐global parcellation of the human cerebral cortex from intrinsic functional connectivity MRI. Cerebral Cortex, 28(9), 3095–3114. 10.1093/cercor/bhx179 28981612 PMC6095216

[brb33015-bib-0051] Shah, L. M. , Cramer, J. A. , Ferguson, M. A. , Birn, R. M. , & Anderson, J. S. (2016). Reliability and reproducibility of individual differences in functional connectivity acquired during task and resting state. Brain and Behavior, 6(5), e00456. 10.1002/brb3.456 27069771 PMC4814225

[brb33015-bib-0052] Shaw, J. C. (1981). An introduction to the coherence function and its use in EEG signal analysis. Journal of Medical Engineering & Technology, 5(6), 279–288. 10.3109/03091908109009362 7328624

[brb33015-bib-0053] Shine, J. M. , Bissett, P. G. , Bell, P. T. , Koyejo, O. , Balsters, J. H. , Gorgolewski, K. J. , & Poldrack, R. A. (2016). The dynamics of functional brain networks: Integrated network states during cognitive task performance. Neuron, 92(2), 544–554. 10.1016/j.neuron.2016.09.018 27693256 PMC5073034

[brb33015-bib-0054] Silver, N. C. , & Dunlap, W. P. (1987). Averaging correlation coefficients: Should Fisher's z transformation be used? Journal of Applied Psychology, 72(1), 146–148. 10.1037/0021-9010.72.1.146

[brb33015-bib-0055] Smith, S. M. , Fox, P. T. , Miller, K. L. , Glahn, D. C. , Fox, P. M. , Mackay, C. E. , & Beckmann, C. F. (2009). Correspondence of the brain's functional architecture during activation and rest. Proceedings of the National Academy of Sciences of the United States of America, 106(31), 13040–13045. 10.1073/pnas.0905267106 19620724 PMC2722273

[brb33015-bib-0056] Takamura, T. , & Hanakawa, T. (2017). Clinical utility of resting‐state functional connectivity magnetic resonance imaging for mood and cognitive disorders. Journal of Neural Transmission, 124(7), 821–839. 10.1007/s00702-017-1710-2 28337552

[brb33015-bib-0057] Tambini, A. , Rimmele, U. , Phelps, E. A. , & Davachi, L. (2017). Emotional brain states carry over and enhance future memory formation. Nature Neuroscience, 20(2), 271–278. 10.1038/nn.4468 28024158 PMC12977101

[brb33015-bib-0058] Toschi, N. , Riccelli, R. , Indovina, I. , Terracciano, A. , & Passamonti, L. (2018). Functional connectome of the five‐factor model of personality. Personality Neuroscience, 1, E2. 10.1017/pen.2017.2 30294715 PMC6171528

[brb33015-bib-0059] Tracy, J. I. , & Doucet, G. E. (2015). Resting‐state functional connectivity in epilepsy. Current Opinion in Neurology, 28(2), 158–165. 10.1097/WCO.0000000000000178 25734954

[brb33015-bib-0060] Tucker, D. M. , Roth, D. L. , & Bair, T. B. (1986). Functional connections among cortical regions: Topography of EEG coherence. Electroencephalography and Clinical Neurophysiology, 63(3), 242–250. 10.1016/0013-4694(86)90092-1 2419082

[brb33015-bib-0061] Van Den Heuvel, M. P. , Mandl, R. C. W. , Kahn, R. S. , & Hulshoff Pol, H. E. (2009). Functionally linked resting‐state networks reflect the underlying structural connectivity architecture of the human brain. Human Brain Mapping, 30(10), 3127–3141. 10.1002/hbm.20737 19235882 PMC6870902

[brb33015-bib-0062] Wang, D. , Buckner, R. L. , Fox, M. D. , Holt, D. J. , Holmes, A. J. , Stoecklein, S. , & Liu, H. (2015). Parcellating cortical functional networks in individuals. Nature Neuroscience, 18(12), 1853–1860. 10.1038/nn.4164 26551545 PMC4661084

[brb33015-bib-0063] Wang, L. , Su, L. , Shen, H. , & Hu, D. (2012). Decoding lifespan changes of the human brain using resting‐state functional connectivity MRI. PLoS ONE, 7(8), e44530. 10.1371/journal.pone.0044530 22952990 PMC3431403

[brb33015-bib-0064] Yeo, B. T. , Krienen, F. M. , Sepulcre, J. , Sabuncu, M. R. , Lashkari, D. , Hollinshead, M. , & Buckner, R. L. (2011). The organization of the human cerebral cortex estimated by intrinsic functional connectivity. Journal of Neurophysiology, 106(3), 1125–1165. 10.1152/jn.00338.2011 21653723 PMC3174820

[brb33015-bib-0065] Zeithamova, D. , de Araujo Sanchez, M. A. , & Adke, A. (2017). Trial timing and pattern‐information analyses of fMRI data. Neuroimage, 153, 221–231. 10.1016/j.neuroimage.2017.04.025 28411155

[brb33015-bib-0066] Zuo, X. N. , Kelly, C. , Adelstein, J. S. , Klein, D. F. , Castellanos, F. X. , & Milham, M. P. (2010). Reliable intrinsic connectivity networks: Test‐retest evaluation using ICA and dual regression approach. Neuroimage, 49(3), 2163–2177. 10.1016/j.neuroimage.2009.10.080 19896537 PMC2877508

